# Supplementary material for: The genome sequence of Pandarus bicolor Leach, 1816 (Siphonostomatoida: Pandaridae)
Source: Wellcome Open Res. 2026 Aug 25;11:544. [Version 1] doi: 10.12688/wellcomeopenres.27381.1 (PMC13717174; doi:10.12688/wellcomeopenres.27381.1)
Supplement: Peer review history

# Reviewer report: Reviewer response for version 1

**DOI:** 10.21956/wellcomeopenres.30136.r171883

**Published:** 2026-10-28

**Authors:** Michael Dondrup

**Affiliations:** 1 ELIXIR Norway, Computational Biology Unit, Department of Informatics, University of Bergen, Thormøhlensgate 55, Bergen, Norway

Adkins *et al.* report a chromosome-level assembly, including an alternate haplotype, of the crustacean ectoparasite *Pandarus bicolor*. Parasitic copepods are still a scarcely represented group in public genome databases. The methods used are highly standardized, and the resulting sequence appears complete and of high quality. The authors have performed rigorous quality control, and the metrics meet or exceed the benchmark criteria set by the Earth BioGenome project. 

 I have a few comments that may help to improve the article. 

 The Background section is very brief. In particular, for readers not deeply familiar with copepods and sea lice, a short introduction to this species’ ecology would help. Mentioning phylogenetic position within the Siphonostomatoida and their relation to the Caligidae, host associations, life cycle, and the lack of genomes from non-Caligidae parasitic copepods would help to put this work into context.

 The most important limitation of this genome is currently the lack of an annotation. While the genome annotation has been planned through the Ensembl pipeline, the authors should provide an update with informative descriptors of gene content.  

 An estimate of repeat content is given based only on k-mer frequencies. K-mer-based analyses may underestimate the real repeat content because they mainly capture high-copy, near-identical repeats, not diverged interspersed elements. Recent analyses of other sea lice genomes (e.g., *Lepeophtheirus salmonis* and *Caligus* species) have found a large proportion of interspersed repeats (~60%). A preliminary analysis using a de novo RepeatModeler library and a subsequent RepeatMasker run revealed approximately 68% repeat content in the presented P. bicolor assembly. The authors should attempt to assess and annotate repeat content in the assembly using a dedicated repeat-finding pipeline.

 In the section “Sample acquisition and DNA barcoding”, the authors mention that they collected three adult specimens. The specimen depicted in Figure 1 is female; if possible, the authors should specify the sex of the specimens used. This affects the potential to identify sex chromosomes, and it is also relevant if the sequenced individuals differ. 

 The authors report that 94.01% of the assembly could be assigned to chromosomes. It would be interesting to know whether the arthropod telomere motif (or any other telomere) can be detected. The authors should scan the genome for telomeric repeats, e.g., using tidk.

 Please add a scale bar to Figure 1.

Are sufficient details of methods and materials provided to allow replication by others?

Partly

Is the rationale for creating the dataset(s) clearly described?

No

Are the datasets clearly presented in a useable and accessible format?

Partly

Are the protocols appropriate and is the work technically sound?

Yes

Reviewer Expertise:

Genomics, bioinformatics, transcriptomics, sea lice research

I confirm that I have read this submission and believe that I have an appropriate level of expertise to confirm that it is of an acceptable scientific standard, however I have significant reservations, as outlined above.

# Reviewer report: Reviewer response for version 1

**DOI:** 10.21956/wellcomeopenres.30136.r171882

**Published:** 2026-10-22

**Authors:** Simen Sandve

**Affiliations:** 1 Norwegian University of Life Sciences, Ås, Norway

This manuscript is a genome report accompanying a genome sequence of *Pandarus bicolor* which has been part of the Darwin Tree of Life project.

 The report is well written and contains all the information needed to reproduce the sampling, lab-work and analyses. 

 I have one minor comment: The y-axis label in Figure 6 could be more informative, for example “Mean read coverage (×, log scale)”.”

Are sufficient details of methods and materials provided to allow replication by others?

Yes

Is the rationale for creating the dataset(s) clearly described?

Yes

Are the datasets clearly presented in a useable and accessible format?

Yes

Are the protocols appropriate and is the work technically sound?

Yes

Reviewer Expertise:

Genomics and genome evolution

I confirm that I have read this submission and believe that I have an appropriate level of expertise to confirm that it is of an acceptable scientific standard.

# Reviewer report: Reviewer response for version 1

**DOI:** 10.21956/wellcomeopenres.30136.r168203

**Published:** 2026-10-12

**Authors:** Kevin M. Kocot

**Affiliations:** 1 University of Alabama, Tuscaloosa, Alabama, USA

In this study, Adkins et al. report the chromosome-level genome of the parasitic copepod *Pandarus bicolor*. The methods are state of the art and the genome is assembly seems to be rather complete and of very high quality. I have only a few minor comments that I think would improve the manuscript and make it easer for readers to understand what was done.

 Copepods are diverse and even veteran carcinologists might not be familiar with this family. I suggest inserting “Copepoda:” before “Siphonostomatoida” – this may help increase readership.

 I think the introduction would be improved by more about the ecological or evolutionary significance of this species. Currently, the rationale for creating the dataset is not clearly described.

 “Leach (1816) described Pandarus bicolor as a parasite of the Tope, then known as *Squalus galeus*.” – I am confused by this statement. Does this shark go by a different name now? If so, I would use the currently accepted name or otherwise please clarify what is meant here.

 Please add a scale bar to Figure 1 or, if that cannot be done with confidence, please at least  indicate the approximate size of the animal in the caption of Figure 1. This animal is bigger than I think most folks would guess.

 “A small sample was dissected from the specimen and stored in ethanol” – Please indicate what tissue was used if recorded.

 There are several DNA barcoding protocols on the page linked to on Protocols.io. Please state which protocol was followed and ideally at least mention which primers were used in the main text.

 Was the COI barcode uploaded to NCBI, BOLD, or another database? I realize the whole mt genome is available now but presumably that sequence was uploaded too?

 DNA concentration (but not volume) is stated but these details are not provided for the RNA.

 How much DNA was used for PacBio library prep?

 Is the BlobDir publicly available?

 I was disappointed to see that no annotation is provided. If that is now available, I would encourage the authors to include it in the manuscript.

Are sufficient details of methods and materials provided to allow replication by others?

Partly

Is the rationale for creating the dataset(s) clearly described?

No

Are the datasets clearly presented in a useable and accessible format?

No

Are the protocols appropriate and is the work technically sound?

Yes

Reviewer Expertise:

Invertebrate biodiversity and systematics

I confirm that I have read this submission and believe that I have an appropriate level of expertise to confirm that it is of an acceptable scientific standard, however I have significant reservations, as outlined above.
